# Quantifying the effects of five rehabilitation training methods on the ability of elderly men to control bowel movements: a finite element analysis study

**DOI:** 10.3389/fbioe.2024.1392448

**Published:** 2024-06-26

**Authors:** Rui Wang, Guangtian Liu, Liwei Jing, Jing Zhang, Yan Ye, Haoran Zhu

**Affiliations:** ^1^ School of Nursing, Capital Medical University, Beijing, China; ^2^ College of Nursing and Rehabilitation, North China University of Science and Technology, Hebei, China

**Keywords:** elderly men, rehabilitation training, muscles, quantification, urinary and defecation control ability, finite element analysis

## Abstract

**Purpose:**

The study aims to develop a finite element model of the pelvic floor and thighs of elderly men to quantitatively assess the impact of different pelvic floor muscle trainings and the urinary and defecation control ability.

**Methods:**

A finite element model of the pelvic floor and thighs of elderly men was constructed based on MRI and CT. Material properties of pelvic floor tissues were assigned through literature review, and the relative changes in waistline, retrovesical angle (RVA) and anorectad angulation (ARA) to quantitatively verify the effectiveness of the model. By changing the material properties of muscles, the study analyzed the muscle strengthening or impairment effects of the five types of rehabilitation training for four types of urination and defecation dysfunction. The changes in four outcome indicators, including the retrovesical angle, anorectad angulation, stress, and strain, were compared.

**Results:**

This study indicates that ARA and RVA approached their normal ranges as material properties changed, indicating an enhancement in the urinary and defecation control ability, particularly through targeted exercises for the levator ani muscle, external anal sphincter, and pelvic floor muscles. This study also emphasizes the effectiveness of personalized rehabilitation programs including biofeedback, exercise training, electrical stimulation, magnetic stimulation, and vibration training and advocates for providing optimized rehabilitation training methods for elderly patients.

**Discussion:**

Based on the results of computational biomechanics, this study provides foundational scientific insights and practical recommendations for rehabilitation training of the elderly’s urinary and defecation control ability, thereby improving their quality of life. In addition, this study also provides new perspectives and potential applications of finite element analysis in elderly men, particularly in evaluating and designing targeted rehabilitation training.

## 1 Introduction

In contemporary society, the aging of the population continues to intensify, and the health problems of the elderly have received increasing attention. The decline in muscle strength and diminished neurological control often experienced with age and frailty lead to conditions such as urinary incontinence, urinary retention, fecal incontinence and constipation (Talasz et al., 2018; Ma and Chen, 2020; Levaillant et al., 2023; Salari et al., 2023), significantly affecting life quality. The resultant emotional distress and increased dependency on caregivers (Bektas et al., 2023), which plays a vital role in determining the care dependence of the elderly and has an impact on social and economic development. It is becoming more and more prominent that how to effectively improve the urinary and defecation control ability of the elderly in the field of rehabilitation medicine.

For the elderly retaining partial urinary and defecation control ability, the five rehabilitation training methods of exercise training, magnetic stimulation, electrical stimulation, vibration stimulation and biofeedback, which are validated for enhancing the muscles strength in the pelvic floor, abdomen, back, and hips, thereby improving control bowel movements (Mazur-Bialy et al., 2020; Alouini et al., 2022). However, different rehabilitation training methods may have different effects on the urinary and defecation control ability, it is necessary to delineate the quantitative relationship between different rehabilitation training methods and urinary and defecation control ability and to understand the underlying mechanism to provide guidance for clinical rehabilitation practices. Finite element analysis (FEA), a critical computational tool, is considered a technique for simulating the mechanical properties of an object by dividing it into discrete elements and creating a numerical calculation model to represent its behavior (Chen et al., 2010). In recent years, it is widely used in the urethral support function (Peng et al., 2016), the occurrence of levator ani muscle injury and pelvic floor disease during vaginal delivery (Zhou et al., 2020), and the mechanical mechanism of posterior vaginal prolapse (Qiu, 2017). By establishing finite element models, it can simulate the biomechanical behavior of human tissues and organs and analyze the biomechanics of complex systems quantitatively. It is a method that provides a new idea for the evaluation of the effects of rehabilitation training (Yang et al., 2024). This approach underpins the development of models to simulate elderly men’s pelvic floor structures, enhancing our understanding of urinary and defecation control ability mechanisms (Zhang and Yao, 2015).

This study aims to develop a finite element model to simulate five distinct rehabilitation training methods. By changing the values of material properties proportionally, the strengthening and impairment of muscle capacity are simulated. Meanwhile, the defecation ability of the elderly is reflected through the retrovesical angle (RVA) and anorectad angulation (ARA). By comparing the changes in defecation outcome indicators under various parameter settings, the mechanism and relationship between the rehabilitation training methods and urinary and defecation control ability is quantified. This provides precise and effective guidance for the rehabilitation training of the elderly, thereby improving their quality of life.

## 2 Methods

### 2.1 Participant

**FIGURE 1:**
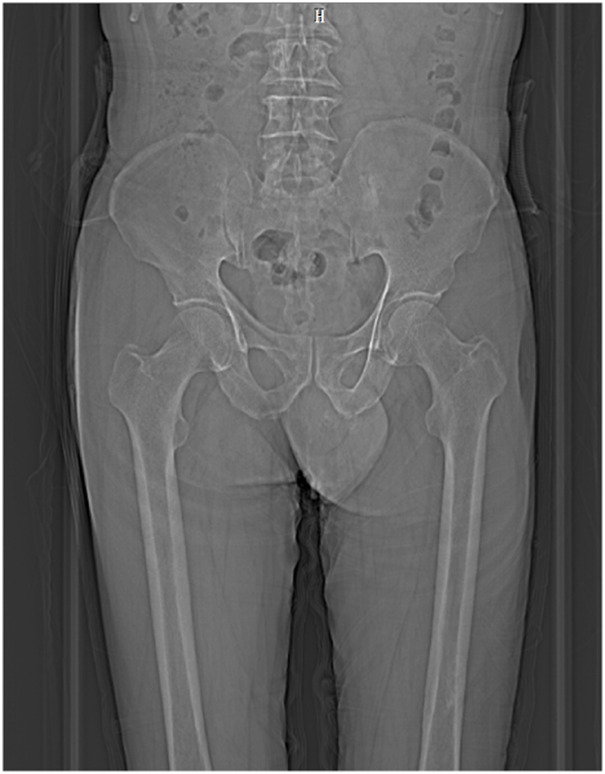
CT image.

**FIGURE 2:**
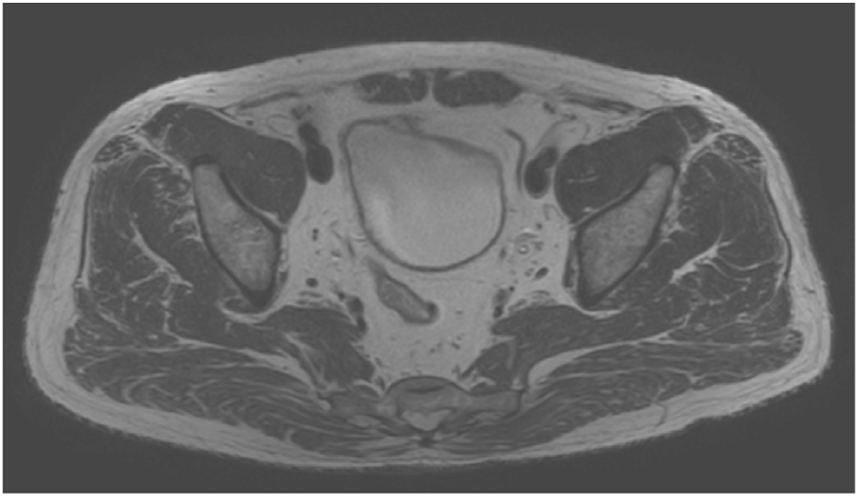
Static MRI image.

**FIGURE 3:**
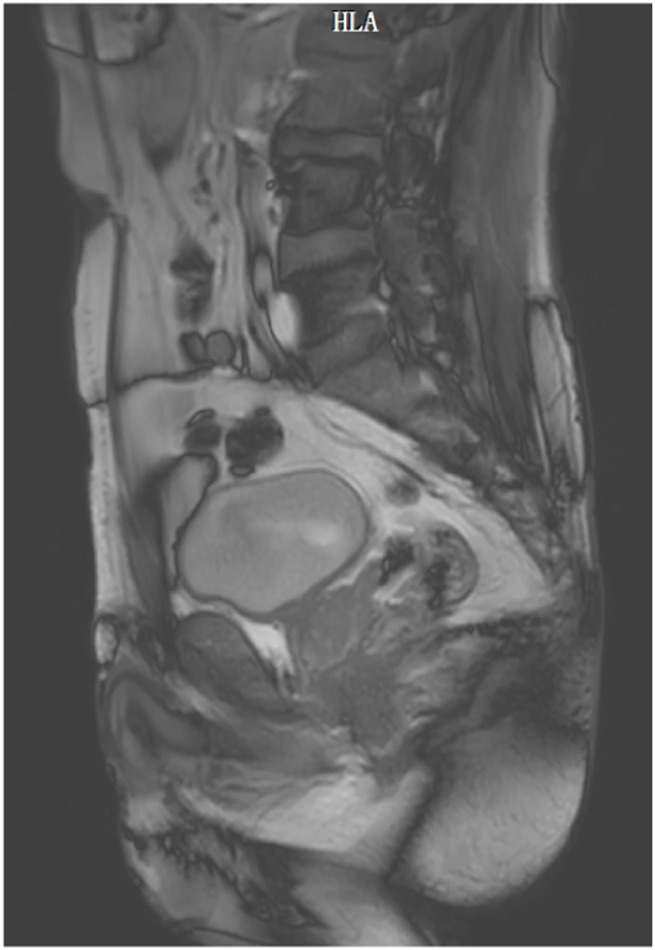
Dynamic MRI image.

The implementation of this study complied with relevant ethical requirements (ethical approval number: Linyanshen [2023] No.079 and Z2024SY007). Our study has been registered in the Chinese Clinical Trial Registry Center (ChiCTR2400080749) and our research protocol has been published online (Wang et al., 2024). The pelvic floor medical imaging data collection participant is a 61-year-old Chinese old adult with a body mass index (BMI) of 25.4 kg/m^2^, good physical health, normal daily living ability, normal cognitive ability, normal communication ability, and normal pelvic floor medical imaging data. The participant’s bowel function was normal, and there was no history of defecation dysfunction or surgery. Participant signed informed consent forms before scanning medical images. During the scan, participant was placed in a supine position with his hands on top of head and legs together and straightened. Scanning started from the highest point of the participant’s iliac crest, CT scan to the knee joint, and MRI scan to 1 cm of the perineum. Radiologists used a 64-slice spiral scanning dual-source CT machine produced by Siemens of Germany and a 3.0T magnetic resonance scanner produced by Philips of the Netherlands to scan, obtain pelvic floor scan data and store it in DICOM format. Among them, CT was scanned with a slice thickness of 0.5 mm and a resolution of 512 × 512, with a total of 598 images, as shown in Figure 1. Static MRI was scanned with 3D-PelvicVIEW-T2, TR 1250 ms, TE 151 ms, field of view 400 cm, continuous 1.0 mm slices Thickness-free volumetric scanning, a total of 400 images, as shown in Figure 2. After the static MRI scan, the participant was asked to perform resting maneuvers, Kegel maneuvers, relaxation maneuvers, and Valsalva maneuvers in order, and at the same time, dynamic MRI scans were performed. Dynamic MRI used the BTFE_Sag_Dyn sequence along the sagittal plane, with a slice thickness of 8 mm and a frame rate of 9–10 frames/s, with a total of 150 images, as shown in Figure 3. For inclusion and exclusion criteria for the participant and the specific data collection methods, please refer to our previous research protocol (Wang et al., 2024).

A composite finite element model of the participant’s pelvic floor, abdomen, back and hip was established through medical imaging data, in which the bone geometry model was through CT images and the soft tissue geometry model was through MRI images. First, the segmentation was performed under the guidance of experienced pelvic floor radiologists. We used the medical modeling software Mimics to reconstruct a relatively rough model, and then used the smoothing function in the Mimics software to beautify it, reduce the angle of points or edges, make the edges smoother, smooth the 3D image, and then the accurate model is imported into Ansys software for numerical simulation calculations.

### 2.2 Material properties

Since this study focuses on the mechanical analysis results of muscles related to urination and defecation, the material properties of the bladder, urethra, rectum, external anal sphincter, external urethral sphincter, and levator ani muscle were analyzed using Yeoh and Mooney-Rivlin hyperelastic structures. For simulation, Hooke linear elastic structures were used for other organs, muscles and fat of the pelvic floor, and viscoelastic structures of the standard linear solid model and hooke were used for the abdominal, back and hip muscles. The pelvis was modeled as a rigid body and fixed structure, considering that its stiffness is much higher than that of soft tissue and therefore its deformation is negligible under normal pelvic function. The finite element type is mainly tetrahedron, supplemented by hexahedron. When setting the material properties in the model, we retrieved existing material data in previous studies (Lee et al., 2005; Boubaker et al., 2009; Zhang et al., 2009; Rao et al., 2010; Venugopala Rao et al., 2010; Wein, 2011; Larson et al., 2012; Chen, 2014; Sichting et al., 2014; Brandão et al., 2015; Chen et al., 2015; Krcmar et al., 2015; Samavati et al., 2015; Sun, 2015; He, 2016; Ma et al., 2016; Peng et al., 2016; Silva et al., 2016; Dias et al., 2017; Li et al., 2017; Liu, 2017; Ricci et al., 2018; Guo et al., 2019; Li et al., 2019; Ma, 2019; Wu, 2019; Zhou et al., 2020; Xu et al., 2021; Xuan et al., 2021; Li, 2022; Yao et al., 2022; Zong, 2022), summarized the mechanical parameters recognized in the literature, and obtained the material properties of the research object in this study. Because the literature shows that there are differences in the mechanical performance characteristics of old and young pelvic soft tissues, and the material properties may change (Chantereau et al., 2014), we made appropriate adjustments on the of material properties, and we verified whether the data conditioning is appropriate by completing the verification steps of the finite element model, the specific parameters are shown in Table 1.

**TABLE 1:** Material properties of various parts in the model.

No.	Anatomical element	Material constants		Structures	Constitutive models
		In the literature	After adjustment		
1	bladder	C_10_ = 0.071 C_20_ = 0.202 C_30_ = 0.048 (Chen et al., 2015; Li, 2022)	C_10_ = 0.061 C_20_ = 0.202 C_30_ = 0.048	hyperelastic structures (Venugopala Rao et al., 2010; Chen et al., 2015)	Yeoh (Chen et al., 2015; Li, 2022)
2	urethra		C_10_ = 0.071 C_20_ = 0.282 C_30_ = 0.048		
3	rectum	C_10_ = 0.088 C_20_ = 3.092 C_30_ = 2.871 (Li, 2022)	C_10_ = 0.082 C_20_ = 3.092 C_30_ = 2.871		
4	prostate	young’s module 6 MPa (Boubaker et al., 2009; Samavati et al., 2015) Poisson’s ratio 0.495 (Boubaker et al., 2009; Samavati et al., 2015)	young’s module 5.8 MPa Poisson’s ratio 0.495	linear elastic structures (Dias et al., 2017)	Hooke (Dias et al., 2017)
5	hipbone	young’s module 15244 MPa (Sichting et al., 2014) Poisson’s ratio 0.3 (Sichting et al., 2014; He, 2016; Peng et al., 2016; Ricci et al., 2018; Guo et al., 2019; Zong, 2022)	No change	rigid body (Brandão et al., 2015; Xu et al., 2021)	
6	sacrum	young’s module 16262 MPa (Sichting et al., 2014) Poisson’s ratio 0.3 (Sichting et al., 2014; He, 2016; Peng et al., 2016; Ricci et al., 2018; Guo et al., 2019; Zong, 2022)	No change		
7	coccyx	young’s module 11000 MPa (Sichting et al., 2014) Poisson’s ratio 0.3 (Sichting et al., 2014; He, 2016; Peng et al., 2016; Ricci et al., 2018; Guo et al., 2019; Zong, 2022)	No change		
8	femur	young’s module 13500 MPa (Guo et al., 2019) Poisson’s ratio 0.3 (Sichting et al., 2014; He, 2016; Peng et al., 2016; Ricci et al., 2018; Guo et al., 2019; Zong, 2022)	No change		
9	fat	young’s module 0.05 MPa (Dias et al., 2017) Poisson’s ratio 0.49 (Rao et al., 2010; Dias et al., 2017; Ma, 2019)	No change	linear elastic structures (Chen et al., 2010; Rao et al., 2010; Dias et al., 2017)	Hooke (Chen et al., 2010; Rao et al., 2010; Dias et al., 2017)
10	bulbospongiosus muscle	young’s module 2.4 MPa (Zhang et al., 2009; Chen et al., 2010; Rao et al., 2010; Dias et al., 2017) Poisson’s ratio 0.49 (Rao et al., 2010; Dias et al., 2017; Ma, 2019)	young’s module 3.4 MPa Poisson’s ratio 0.49		
11	ischiocavernous muscle				
12	superficial transverse perineal muscle, deep transverse perineal muscle				
13	external anal sphincter	C_10_ = 11.8 kPa C_20_ = 5.53e-3 kPa (Silva et al., 2016)	C_10_ = 12.8 kPa C_20_ = 5.53e-3 kPa	hyperelastic structures (Silva et al., 2016; Xuan et al., 2021)	Mooney-Rivlin (Silva et al., 2016)
14	external urethral sphincter				
15	coccygeal muscle	young’s module 2.4 MPa (Zhang et al., 2009; Dias et al., 2017) Poisson’s ratio 0.49 (Dias et al., 2017; Xu et al., 2021)	No change	linear elastic structures (Dias et al., 2017)	Hooke (Dias et al., 2017)
16	levator ani muscle, including pubococcygeus muscle, iliococcygeus muscle, puborectalis muscle	C_10_ = 2.5 KPa C_20_ = 0.625 KPa (Lee et al., 2005; Wu, 2019)	No change	hyperelastic structures (Krcmar et al., 2015)	Mooney-Rivlin (Wu, 2019)
17	obturator internus muscle	young’s module 0.95 MPa (Chen, 2014; Peng et al., 2016; Liu, 2017) Poisson’s ratio 0.45 (Chen, 2014)	No change	viscoelastic materials	Hooke
18	rectus abdominis muscle	young’s module 13.3 MPa (Sun, 2015) Poisson’s ratio 0.45 (Chen, 2014)	No change	viscoelastic materials (Li et al., 2017)	Hooke
19	iliac muscle	young’s module 19 MPa (Li et al., 2017) Poisson’s ratio 0.45 (Chen, 2014)	No change		
20	psoas major muscle	young’s module 13.3 MPa (Sun, 2015) Poisson’s ratio 0.45 (Chen, 2014)	No change		
21	quadriceps femoris muscle				
22	tensor fascia lata muscle				
23	gluteus maximus muscle	young’s module 19 MPa (Li et al., 2017) Poisson’s ratio 0.45 (Chen, 2014)	No change		
24	gluteus medius muscle				
25	piriformis muscle				
26	hamstring muscles	young’s module 13.3 MPa (Sun, 2015) Poisson’s ratio 0.45 (Chen, 2014)	No change		
27	pubic muscle				
28	adductor longus muscle				
29	latissimus dorsi muscle				
30	erector spinae muscle				

### 2.3 Constraint boundaries

The coordinates of the three-dimensional finite element model are set as follows: the x-axis is perpendicular to the sagittal plane of the human body, and the positive direction points to the left; the *y*-axis is perpendicular to the coronal plane of the human body, and the positive direction points backward; the *z*-axis is the longitudinal axis of the human body, and the positive direction points to the head. In the analysis of static structural mechanics, in order to simulate the in-body state of human defecation-related muscles as closely as possible, the bone is set as a rigid body and all nodes are fully constrained. We limited the translation and rotation of bones in the three axes of x, y and z. The tops of the rectus abdominis muscle, psoas major muscle, and erector spinae muscle are fixed. The contact condition between bones and muscles, bladder and prostate, prostate and colon were set to bind.

### 2.4 Model validity verification

In the finite element modeling process, it is very necessary to verify the validity of the finite element model. The methods of finite element model validation usually include geometric shape validation, literature result validation, and experimental result validation (Peng et al., 2023). Peng et al. (2016) conducted a validation study by comparing pelvic floor configurations achieved in computer simulation results with dynamic MRI observations along the midsagittal plane at both rest and maximal Valsalva maneuvers, and we highly recognize this geometric form validation method. Due to the lack of literature results and experimental results of elderly pelvic floor, It is very difficult to verify using methods such as literature results verification and experimental results verification. Therefore, We will compare the Valsalva maneuvers of the elderly during dynamic MRI scanning with the Valsalva maneuvers of the pelvic floor simulated by the finite element model, and compare whether the variety of the three anatomical landmark points of the two are consistent.

The specific process of dynamic MRI scanning is detailed in our previous research protocol (Wang et al., 2024) and we completed the Valsalva maneuvers through simulation of the finite element model. The Valsalva simulation plan’s loading site is the anterior abdominal wall, and in the resting state, E = 0.019 and the load is 0.5 kPa. At moderate tension, E = 0.241 and the load is 4.5 kPa. Under severe tension, E = 0.947 and the load is 5.0 kPa (Wu, 2019), and the direction of application is from the top of the anterior abdominal wall to the direction of the coccyx (Qiu, 2017). During model verification, the three anatomical landmarks include relative changes in waistline, changes in retrovesical angle (RVA) and changes in anorectad angulation (ARA). Among them, the RVA is the angle formed by the median sagittal plane of the bladder floor and the long axis of the urethra, and the ARA is the angle between the longitudinal axis of the anal canal and the posterior wall of the rectum above the levator ani muscle. These two angles are often measured in imaging data of the pelvic floor. Due to the lack of relevant research and guidelines specifying the scope of differences, we consulted relevant biomechanical experts and combined them with clinical situations. If the differences are within 10%, it will be considered that the model construction is more accurate.

### 2.5 Applied loads and simulation plans analysis

After completing the construction and verification of the finite element model, we will conduct specific finite element analysis under different simulation plans. Muscle elastic modulus refers to the ratio of deformation to the force applied on muscles and is a critical parameter for assessing muscle stiffness. Research has shown that rehabilitation exercises such as muscle contraction training and resistance movements can indirectly affect this modulus, resulting in decreased passive stiffness and increased muscle softness and flexibility (de Sousa et al., 2023). Since finite element analysis does not directly simulate rehabilitation training, we adapted simulation to reflect muscle capacity changes after rehabilitation training. So, we simulated a 25%, 50%, 75%, 100% strengthening in muscle capacity, a normal muscle capacity, a 25%, 50%, 75%, 95% impairment in muscle capacity. These adjustments were achieved by varying the material properties’ correlation properties to 1.25x, 1.5x, 1.75x, 2x (for increases), 1x (for baseline), 0.75x, 0.5x, 0.25x, and 0.05x (for decreases), respectively.

Currently, there are five known rehabilitation training methods for improving bowel function including exercise training, biofeedback, electrical stimulation, magnetic stimulation, and vibrational stimulation. Table 2 lists the muscles based on the literature content corresponding to the five types of rehabilitation training for the four types of urination and defecation dysfunction. We found that the five rehabilitation training methods mainly enhance the elderly’s urination and defecation abilities through muscle training, such as external anal sphincter, urethral sphincter, levator ani muscle, pelvic floor muscles, rectus abdominis muscle, hip muscles and erector spinae muscle. When performing finite element analysis, delete tissues and muscles that are weakly related to urination and defecation from the established finite element model, such as obturator internus muscle, skin, fat, pyramidal muscle, tensor fascia lata muscle, pubic muscle and latissimus dorsi muscle.

**TABLE 2:** Simulation plans table.

No.	Simulated rehabilitation trainings	Loading site	Abdominal pressure (kPa)	Outcome indicators
1	anal sphincter training for fecal incontinence	external anal sphincter	0.6	ARA, stress, strain
2	transcutaneous electrical nerve stimulation for urinary retention	urethral sphincter	0.6	RVA, stress, strain
3	a. biofeedback for urinary incontinence b. biofeedback for urinary retention c. Exercise training for fecal incontinence-levator ani activities d. Exercise training for constipation-levator ani exercise	levator ani muscle	0.6	RVA, ARA, stress, strain
4	a. Exercise training for urinary incontinence b. Exercise training for urinary retention	levator ani muscle and urethral sphincter	0.6	RVA, stress, strain
5	a. Magnetic stimulation of constipation b. biofeedback of constipation c. Exercise training for constipation	levator ani muscle and external anal sphincter	0.6	ARA, stress, strain
6	a. Exercise training for urinary incontinence b. Electrical stimulation for urinary incontinence c. Magnetic stimulation for urinary incontinence d. biofeedback for urinary incontinence e. Vibrational stimulation for urinary incontinence f. Exercise training for urinary retention g. Magnetic stimulation for urinary retention h biofeedback for urinary retention i. Electrical stimulation for urinary retention j. Electrical stimulation for fecal incontinence-sacral nerve anterior root electrical stimulation k. biofeedback for fecal incontinence combined with pelvic floor muscle training l. Magnetic stimulation for fecal incontinence m. Exercise training for constipation-pelvic floor muscle training n. Tibial nerve electrical stimulation, sacral nerve electrical stimulation, and transcutaneous acupoint electrical stimulation for constipation o. Magnetic stimulation for constipation p. biofeedback for constipation q. Vibrational stimulation for constipation	pelvic floor muscles	0.6	RVA, ARA, stress, strain
7	magnetic stimulation for fecal incontinence-functional magnetic stimulation	pelvic floor muscles and rectus abdominis muscle and erector spinae muscle	0.6	ARA, stress, strain
8	exercise training for urinary incontinence-hip muscle exercises	pelvic floor muscles and hip muscles	0.6	RVA, stress, strain
9	exercise training for urinary incontinence-suspension exercise training	pelvic floor muscles and rectus abdominis muscle and hip muscles and erector spinae muscle	0.6	RVA, stress, strain

Regarding intra-abdominal pressure, a search of the literature shows that the resting intra-abdominal pressure is 0.4–0.64 kPa (Song et al., 2012), and the abdominal pressure in another literature was 0.665 kPa (Korkmaz and Rogg, 2007), so the abdominal pressure was selected as 0.6 kPa in our study. The main outcome measure of the study are RVA and ARA, which are well-known parameters for evaluating the control ablity of urination and defecation dysfunction (Guo et al., 2015; Zhou and Lai, 2021). The second outcome measure of the study are the von Mises stress cloud map and strain region of the area in the finite element model of elderly men under different simulation plans. The simulation plans table is listed in Table 2. The first two columns list the test number and the simulated rehabilitation trainings, and the third column lists the loading site. The first to ninth simulation plans simulate the muscle strengthening or impairment effects of the five types of rehabilitation training for the four types of urination and defecation dysfunction.

## 3 Results

### 3.1 Finite element model establishment

**FIGURE 4:**
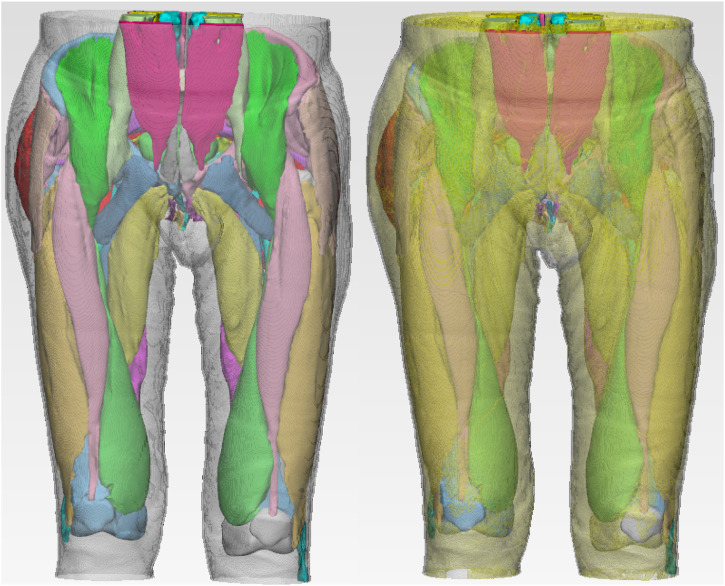
Finite element model of old adult.

**FIGURE 5:**
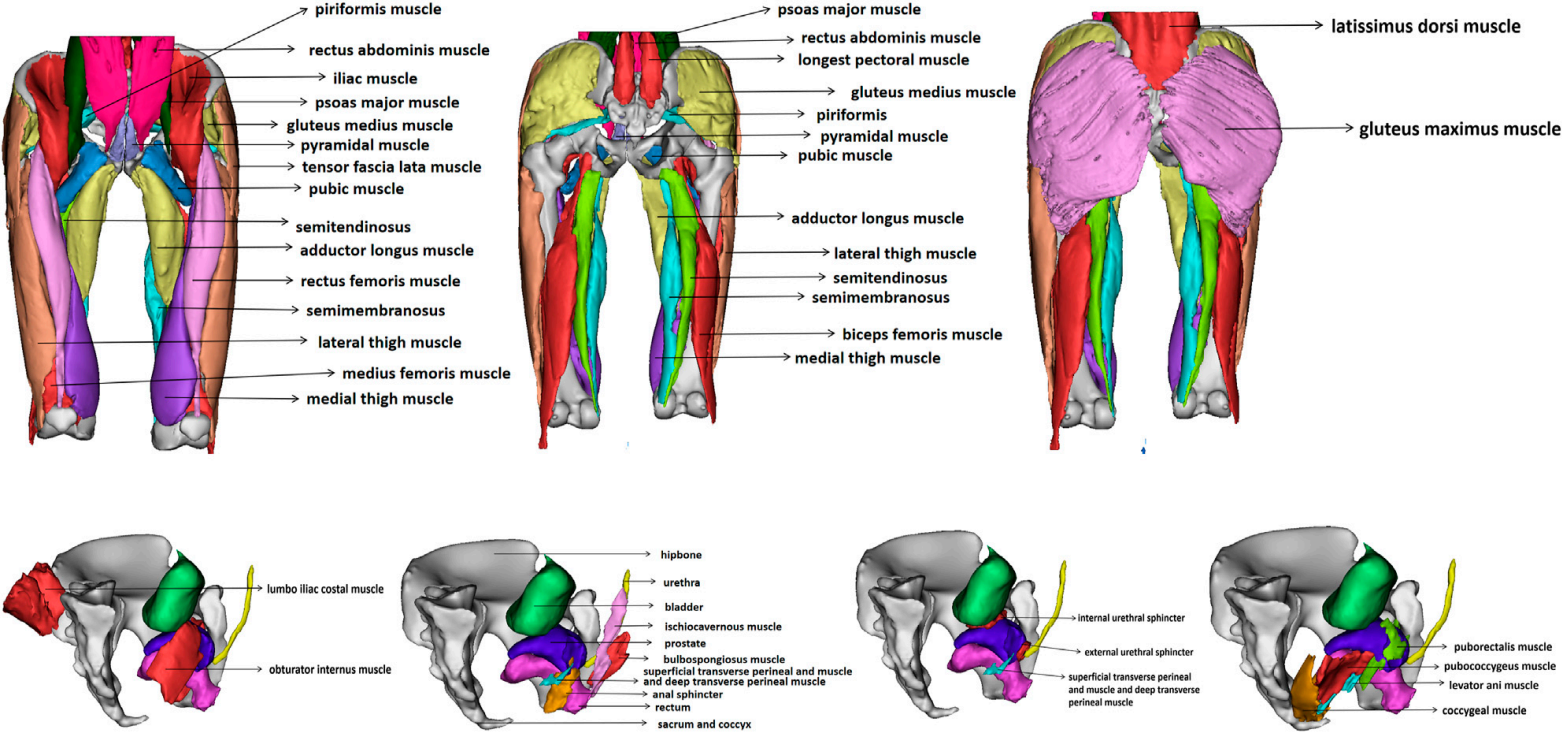
Annotation diagram of different muscle positions of the finite element model.

**FIGURE 6:**
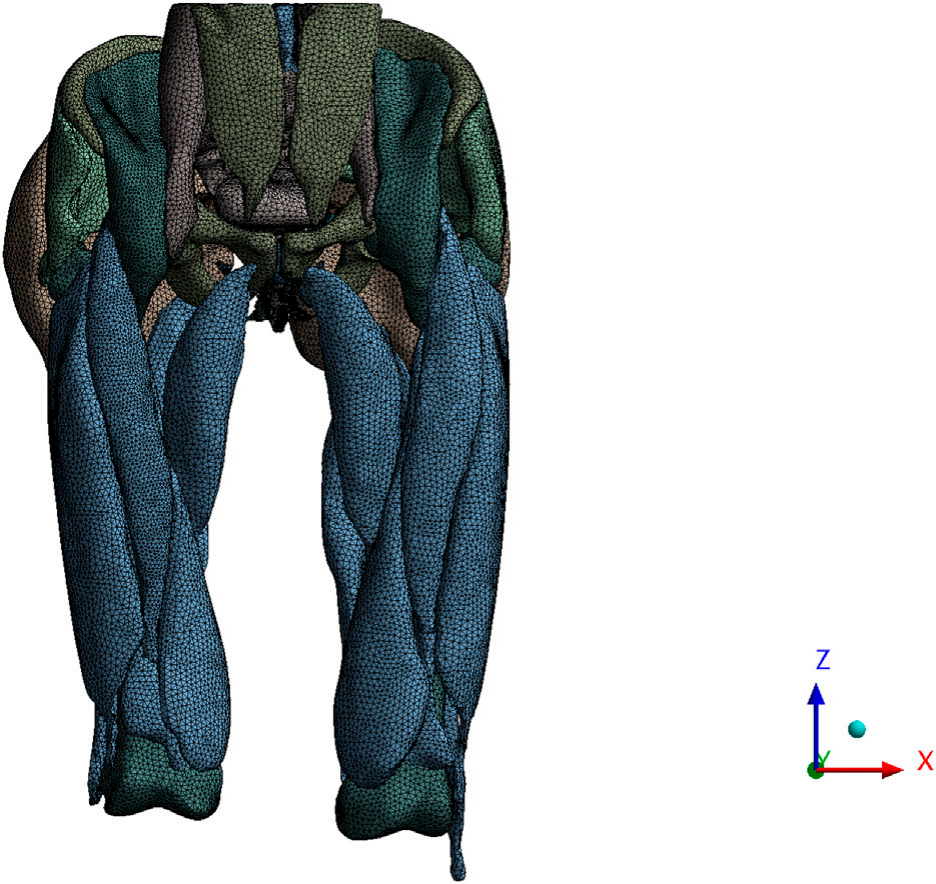
Mesh division of the finite element model.

The finite element model includes the final three-dimensional pelvic floor model including organs including bladder, urethra, rectum and prostate, bones including hipbone, sacrum, coccyx and femur, pelvic floor muscles including bulbocavernosus muscle, ischiocavernosus muscle, superficial transverse perineal muscle, deep transverse perineal muscle, external anal sphincter, external urethral sphincter, coccygeal muscle, pubococcygeus muscle, iliococcygeus muscle, puborectalis muscle and obturator internal muscle, abdominal muscles including rectus abdominis muscle and pyramidal muscle, hip muscles including iliacus muscle, psoas major muscle, rectus femoris muscle, vastus lateralis muscle, vastus intermedius muscle, vastus medialis muscle, tensor fascia lata muscle, gluteus maximus muscle, gluteus medius muscle, piriformis muscle, semitendinosus muscle, semimembranosus muscle, biceps femoris muscle, pubic muscle and adductor longus muscle, back muscles including latissimus dorsi muscle, iliocostalis muscle, longissimus thoracis muscle, skin, fat, a total of 41 structures, 1188,251 nodes and 251,290 elements. Figure 4 shows the finite element model of old adult. Figure 5 is an annotation diagram of different muscle positions of the finite element model. Figure 6 shows the mesh division of the finite element model.

### 3.2 Verification of finite element model

**FIGURE 7:**
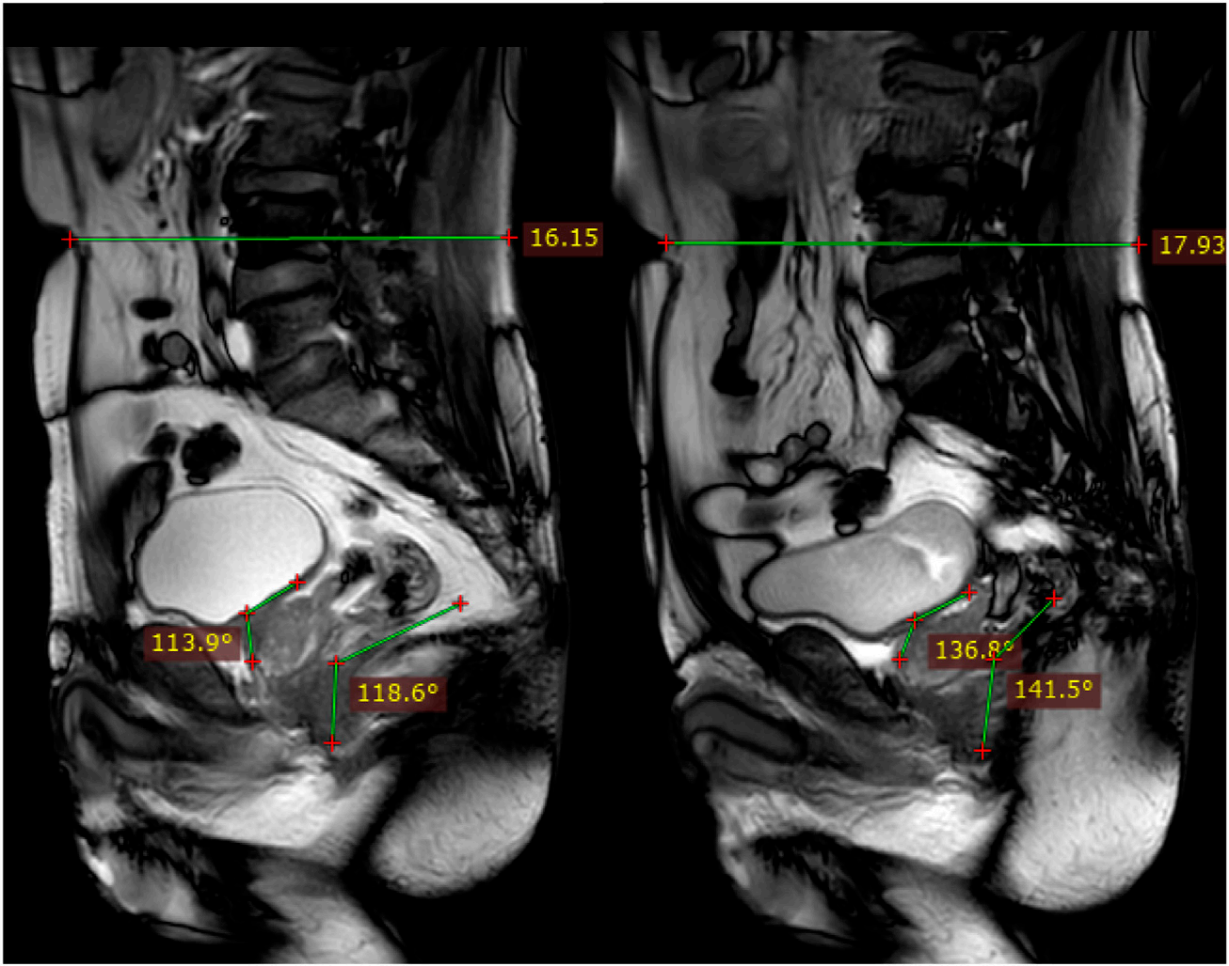
Schematic diagram of measuring relative changes in waistline, RVA, and ARA.

We compared pelvic floor changes achieved along the midsagittal plane in computer simulations with imaging observations of Valsalva maneuvers in dynamic MRI. The results showed that the relative changes in waistline, RVA and ARA are within 3.51%, indicating a high consistency between the simulated situation constructed in the study and the actual pelvic floor situation of the elderly, as shown in Figure 7. The comparison of dynamic MRI and 3D model measurement results are shown in Table 3.

**TABLE 3:** Comparison of dynamic MRI and 3D model measurement results.

Validation indicators		Measurement results		Difference percentage
		Dynamic MRI	3D model	
Waistline	Before Valsalva	161.50 mm	165.72 mm	2.61%
	After Valsalva	179.30 mm	173.01 mm	3.51%
RVA	Before Valsalva	113.9°	115.58°	1.47%
	After Valsalva	136.8°	135.01°	1.31%
ARA	Before Valsalva	118.6°	118.08°	0.44%
	After Valsalva	141.5°	143.44°	1.37%

### 3.3 Finite element simulation plans analysis results

#### 3.3.1 Results of changes in ARA and RVA with material properties

**FIGURE 8:**
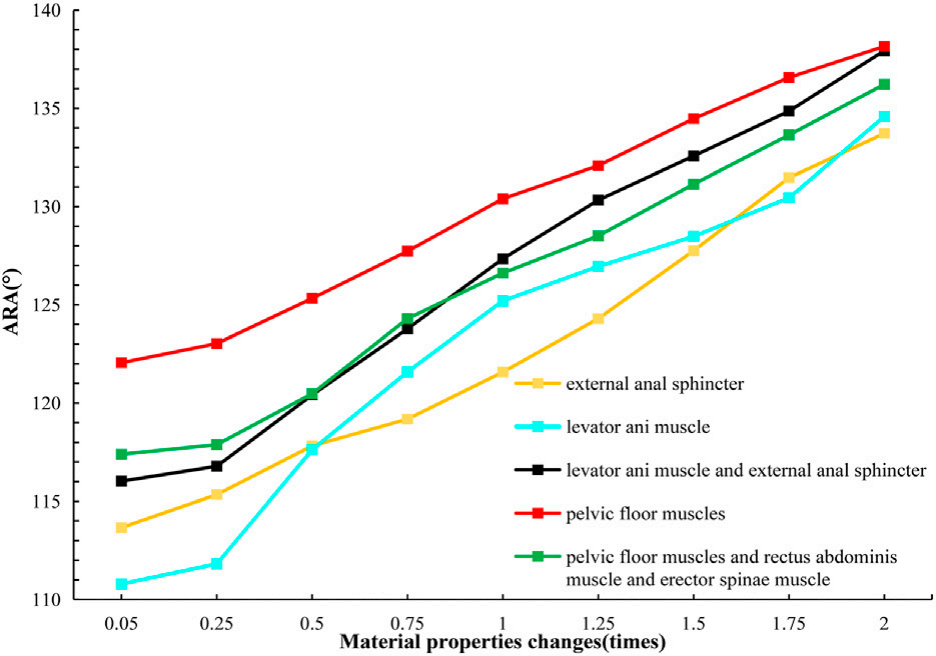
Curve chart of ARA changes with material properties.

**FIGURE 9:**
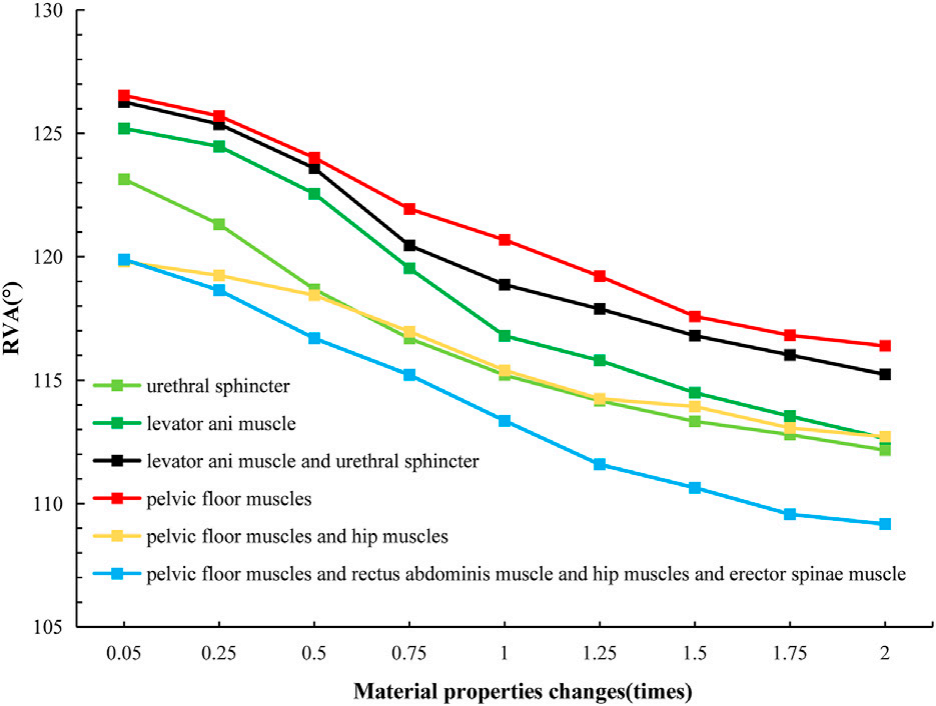
Curve chart of RVA changes with material properties.

We analyzed the numerical changes of ARA and RVA in response to varying material properties respectively, illustrating how these changes correlate with the urinary and defecation abilities of elderly individuals. These relationships are depicted through changes in muscle strength associated with specific defecation and urinary rehabilitation training methods, as shown in Table 4 and 5. Figures 8, 9 present line graphs illustrating the angular responses (ARA and RVA) of five distinct muscle or muscle group combinations to variations in material properties. The x-axis quantifies the changes in material properties as multiplicative factors ranging from 0.05 to 2 times, while the *y*-axis displays the angular response values, with AVA ranging from 110° to 140°and RVA from 105° to 135°. Figure 8 illustrates that with the increase of material properties, the ARA consistently rises under simulation plans 1, 3, 5, 6, and 7. Conversely, as shown in Figure 9, the RVA diminishes with increasing in material properties under simulation plans 2, 3, 4, 6, 8, and 9. The variation in the slope of each line demonstrates the sensitivity of different muscle groups to changes in material properties.

**TABLE 4:** Numerical table of ARA changes with material properties.

Simulation plans	Muscles	Angle	Multiple of material properties									Changes in material properties		
			0.05 times	0.25 times	0.50 times	0.75 times	1.00 times	1.25 times	1.50 times	1.75 times	2.00 times	From 0.05 times to 1 times	From 1 times to 2 times	From 0.05 times to 2 times
No.1	external anal sphincter	ARA	113.66	115.34	117.82	119.18	121.58	124.29	127.75	131.47	133.72	7.92	12.14	20.06
No.3	levator ani muscle	ARA	110.79	111.81	117.63	121.59	125.20	126.95	128.48	130.45	134.58	14.41	9.38	23.79
No.5	levator ani muscle and external anal sphincter	ARA	116.02	116.78	120.42	123.78	127.34	130.33	132.57	134.86	137.93	11.32	10.59	21.91
No.6	pelvic floor muscles	ARA	122.06	123.03	125.33	127.73	130.40	132.08	134.47	136.57	138.16	8.34	7.76	16.1
No.7	pelvic floor muscles and rectus abdominis muscle and erector spinae muscle	ARA	117.39	117.88	120.48	124.29	126.61	128.51	131.14	133.64	136.22	9.22	9.61	18.83

**TABLE 5:** Numerical table of RVA changes with material properties.

Simulation plans	Muscles	Angle	Multiple of material properties									Changes in material properties		
			0.05 times	0.25 times	0.50 times	0.75 times	1.00 times	1.25 times	1.50 times	1.75 times	2.00 times	From 0.05 times to 1 times	From 1 times to 2 times	From 0.05 times to 2times
No.2	urethral sphincter	RVA	123.14	121.32	118.68	116.68	115.21	114.17	113.33	112.79	112.17	−7.93	−3.04	−10.97
No.3	levator ani muscle	RVA	125.20	124.47	122.56	119.53	116.79	115.79	114.49	113.54	112.64	−8.41	−4.15	−12.56
No.4	levator ani muscle and urethral sphincter	RVA	126.27	125.37	123.59	120.46	118.87	117.88	116.80	116.02	115.23	−7.4	−3.64	−11.04
No.6	pelvic floor muscles	RVA	126.54	125.71	124.02	121.94	120.68	119.21	117.58	116.82	116.38	−5.86	−4.3	−10.16
No.8	pelvic floor muscles and hip muscles	RVA	119.8	119.24	118.44	116.97	115.4	114.25	113.93	113.07	112.71	−4.4	−2.69	−7.09
No.9	pelvic floor muscles and rectus abdominis muscle and hip muscles and erector spinae muscle	RVA	119.89	118.64	116.69	115.22	113.36	111.58	110.64	109.57	109.17	−6.53	−4.19	−10.72

#### 3.3.2 Comparative analysis of simulated rehabilitation muscles for constipation and fecal incontinence

**FIGURE 10:**
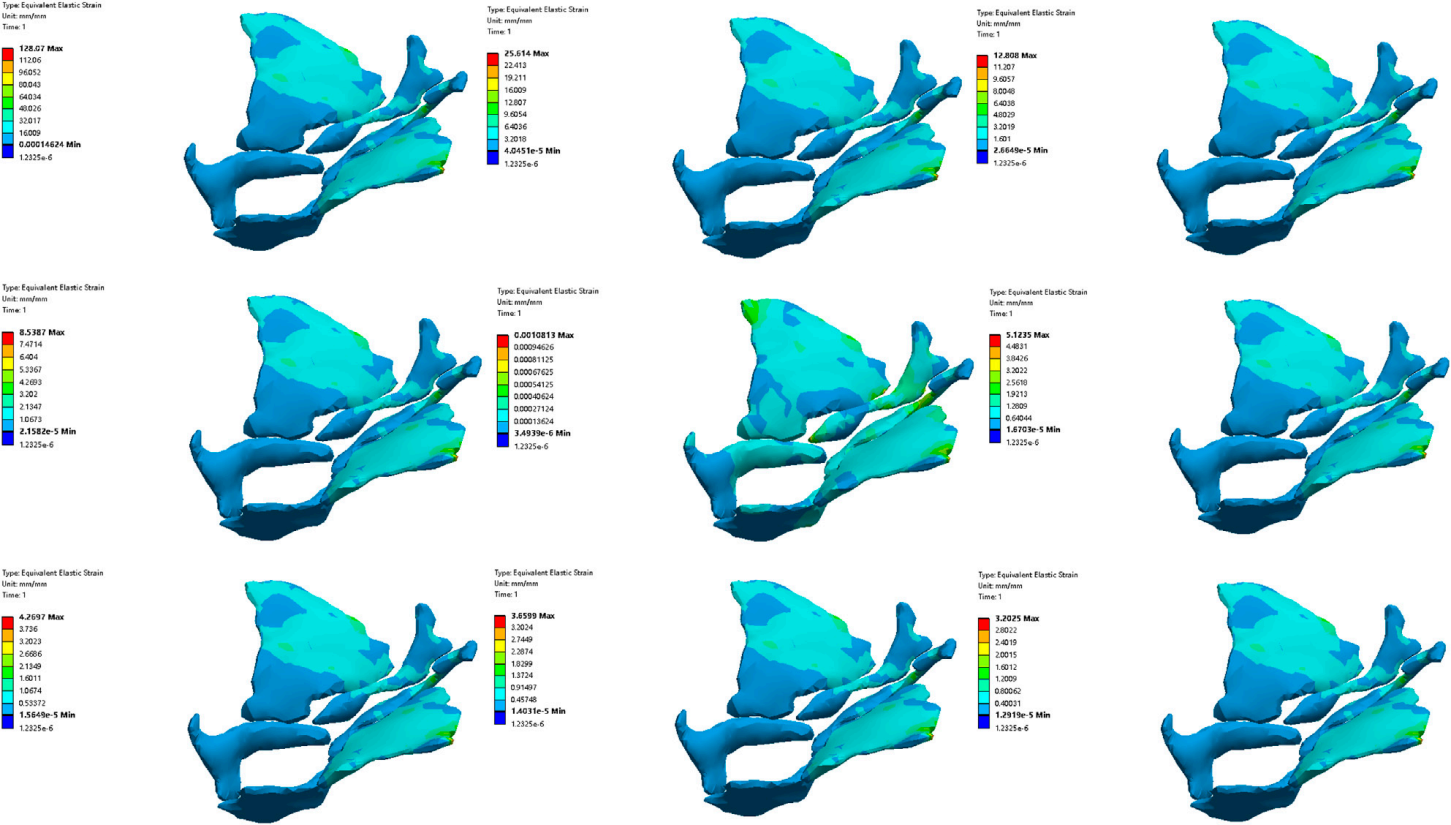
Strain diagram of levator ani muscle in simulation plan No.3.

**FIGURE 11:**
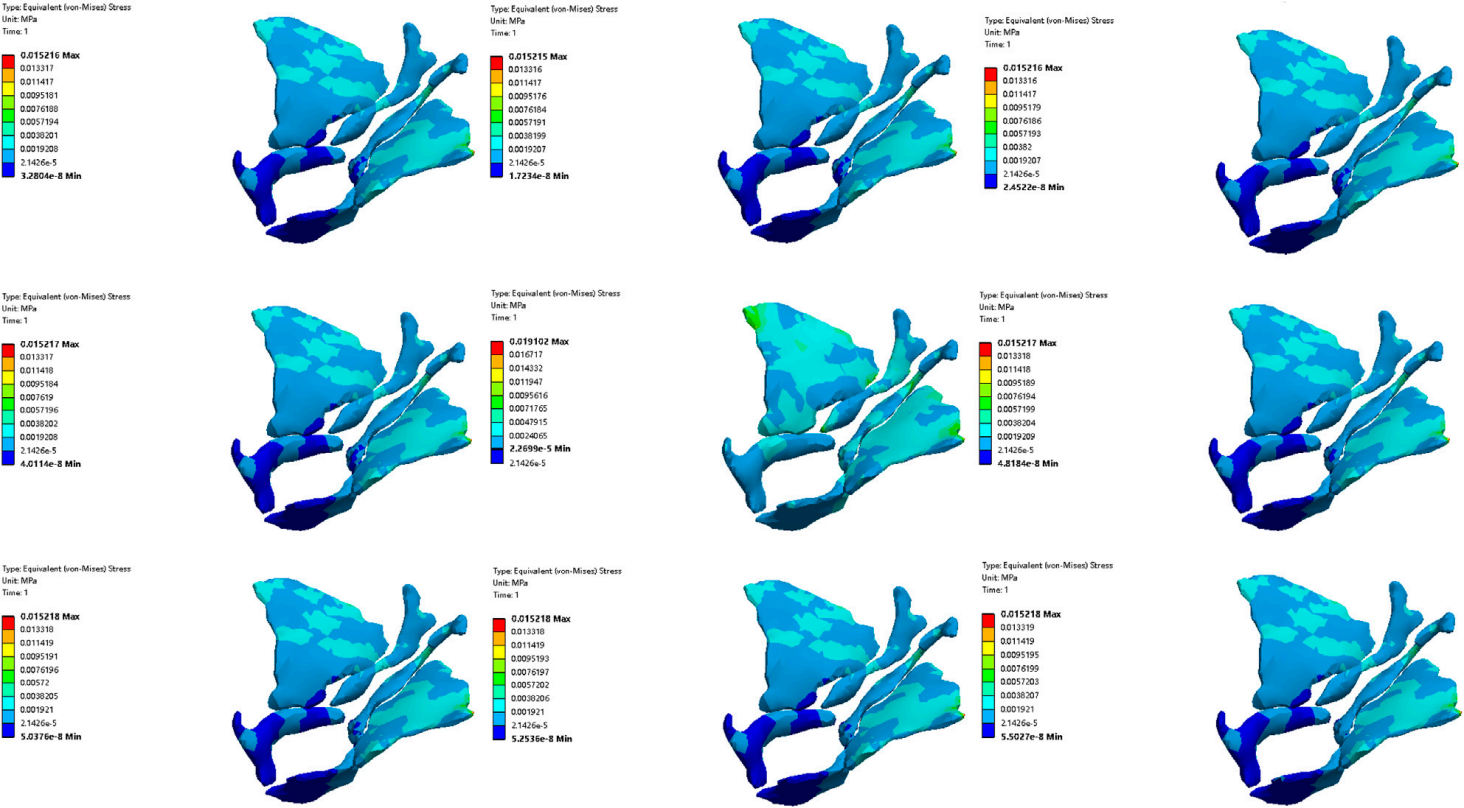
Stress diagram of levator ani muscle in simulation No.3.

**FIGURE 12:**
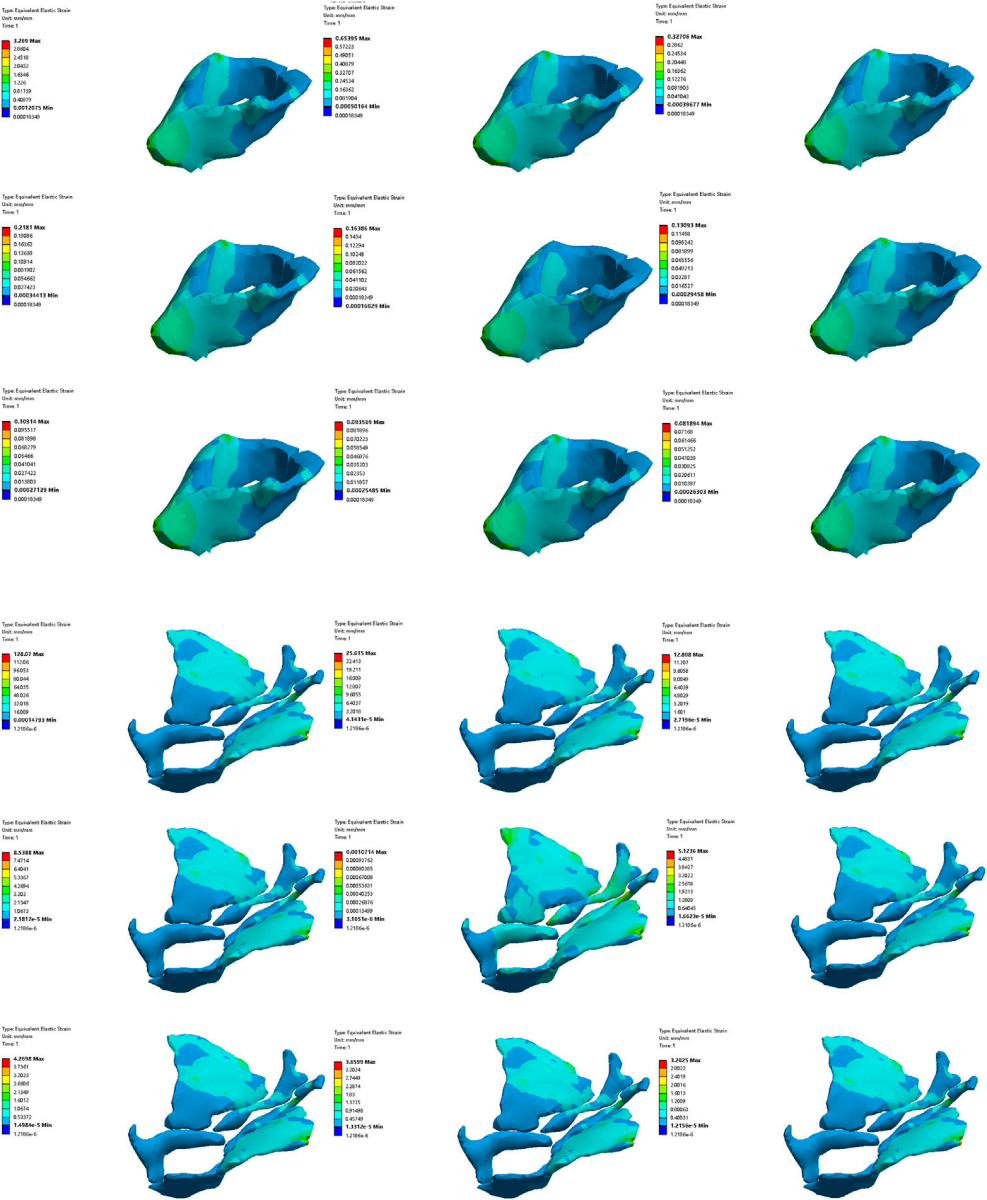
Strain diagram of levator ani muscle and external anal sphincter in simulation No.5.

**FIGURE 13:**
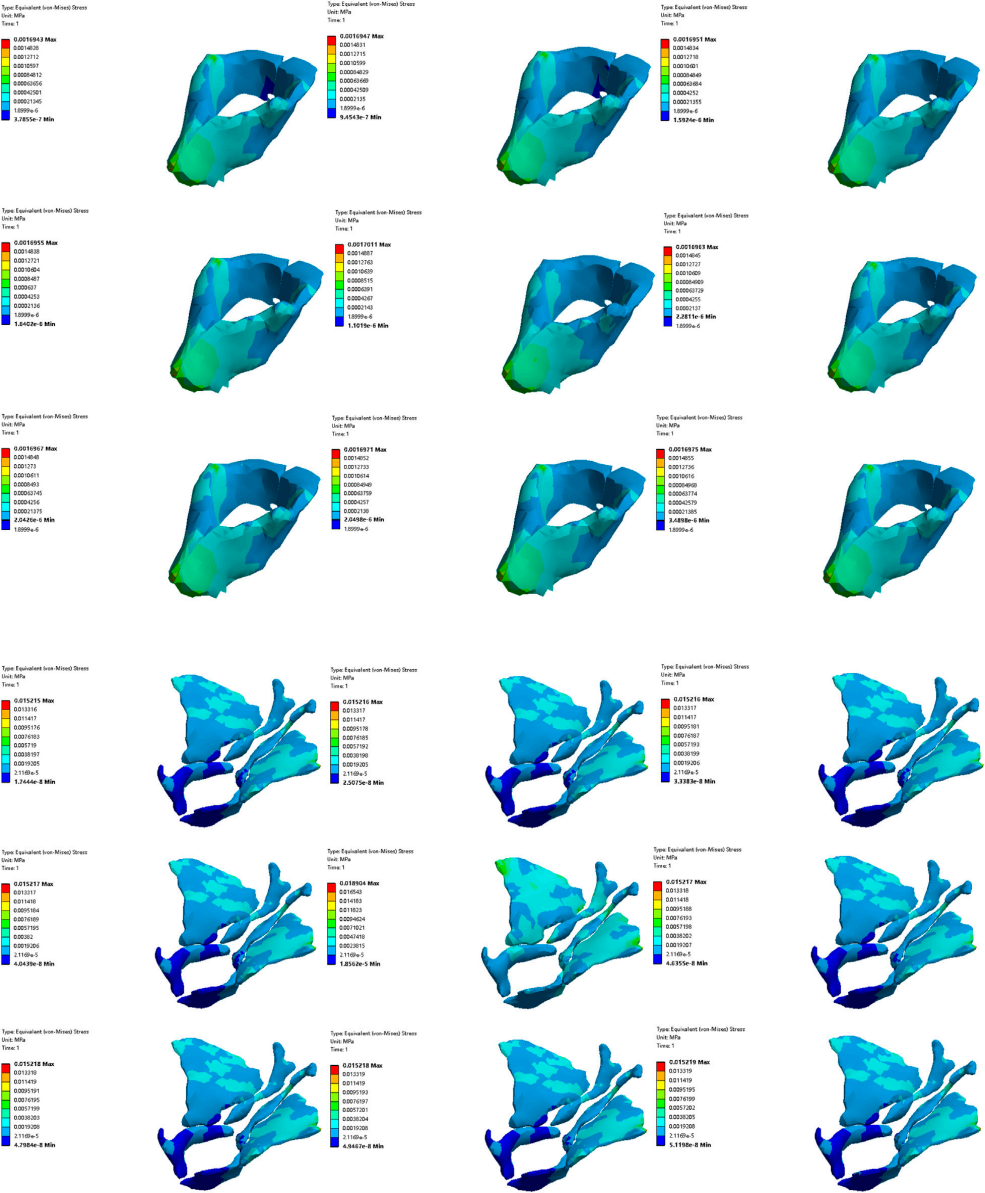
Stress diagram of levator ani muscle and external anal sphincter in simulation No.5.

Simulation plans 3, 5, and 6 were designed to simulate the muscles strengthening and impairment effects on muscles implicated in constipation rehabilitation training. The muscles examined include levator ani muscle, levator ani muscle and external anal sphincter, and pelvic floor muscles. Comparative analysis of improvement of ARA across different simulations, if rehabilitation training gradually changes the material properties of muscles in elderly people from below normal to normal, the training effect of levator ani muscle is better than that of levator ani muscle, external anal sphincter, and pelvic floor muscles in sequence (Figures 10, 11). When the material properties of muscles gradually changes from greater than the normal value to the normal value, the training effect of levator ani muscle and external anal sphincter is better than levator ani muscle and pelvic floor muscles in sequence (Figures 12, 13).

**FIGURE 14:**
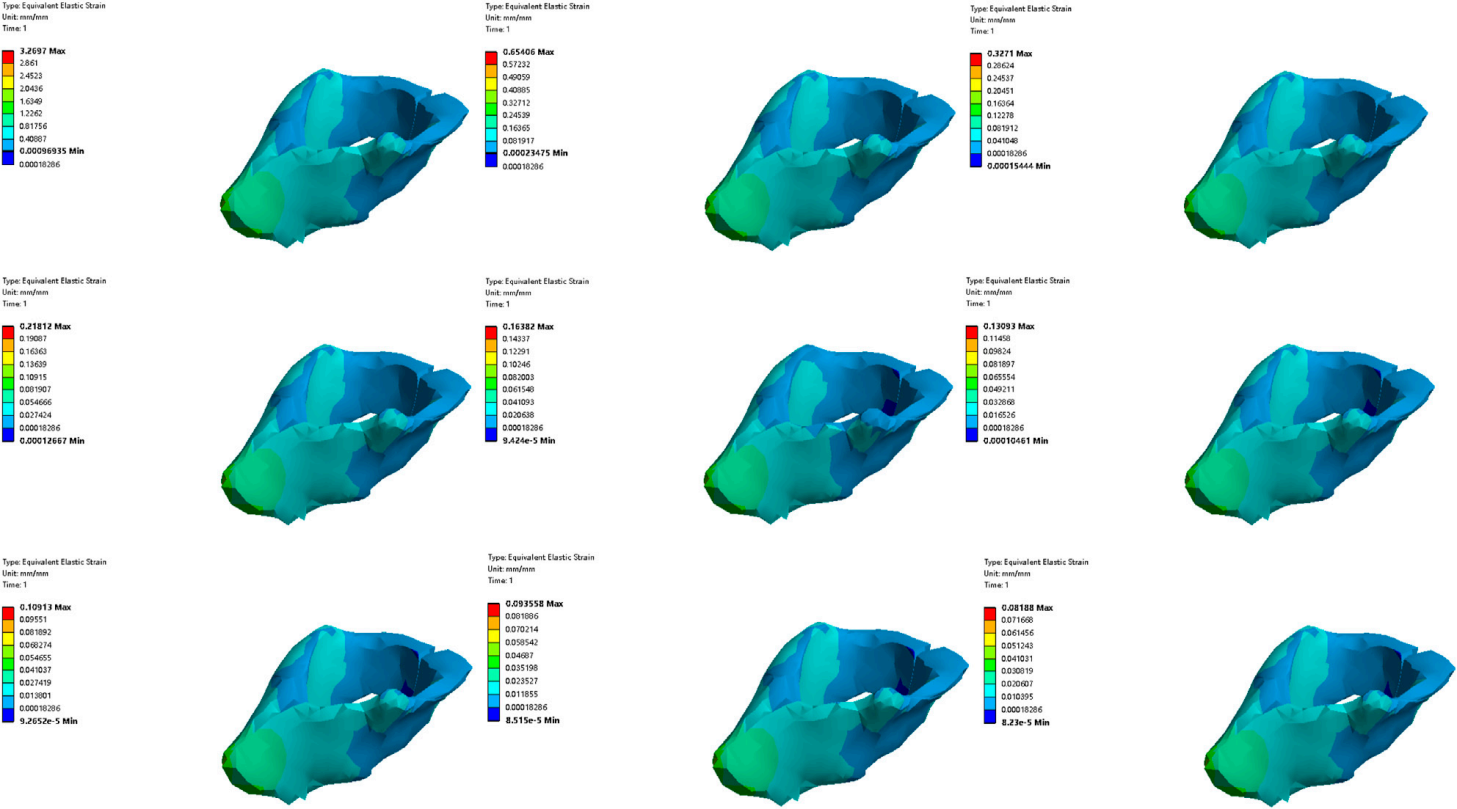
Strain diagram of external anal sphincter in simulation No.1.

**FIGURE 15:**
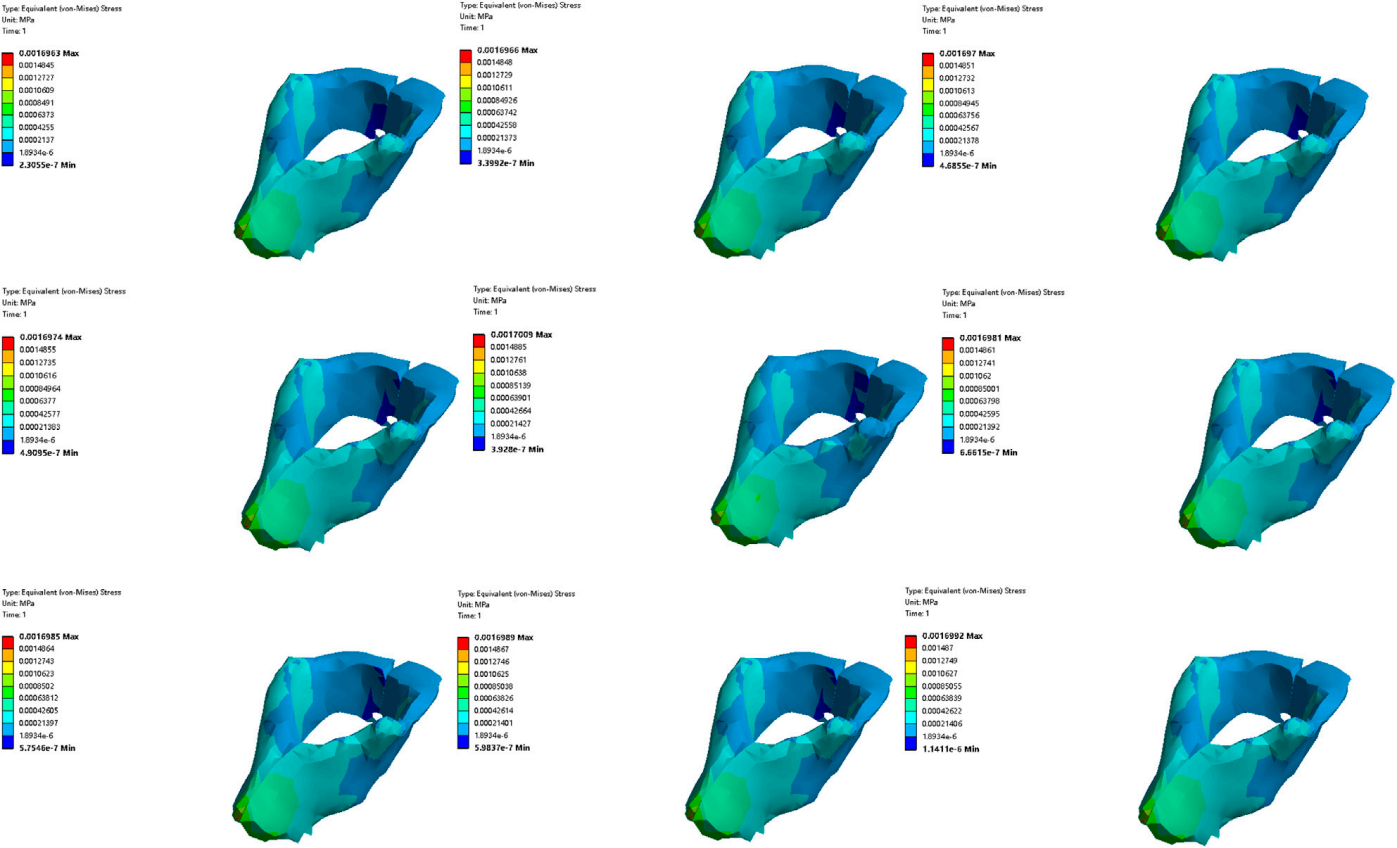
Stress diagram of external anal sphincter in simulation No.1.

Simulation plans 1, 3, 6, and 7 were designed to simulate the muscles strengthening and impairment effects of fecal incontinence rehabilitation training, among which the muscles included external anal sphincter, levator ani muscle, pelvic floor muscles, pelvic floor muscles and rectus abdominis muscle and erector spinae muscle. By comparing the improvement degree of ARA under different simulations, when rehabilitation training gradually changes the material properties of muscles in elderly people from less than normal to normal the training effect of levator ani muscle is better than that of pelvic floor muscles and rectus abdominis muscle and erector spinae muscle, pelvic floor muscles, and external anal sphincter, sequentially (Figures 10, 11). As the material properties of muscles gradually changes from approach normalcy from above-normal levels, the training effect of external anal sphincter is better than that of pelvic floor muscles and rectus abdominis muscle and erector spinae muscle, levator ani muscle, and pelvic floor muscles, respectively (Figures 14, 15).

It is noteworthy that regardless of muscle material properties in elderly people, training the levator ani muscle alone yields better outcomes than training it as part of the pelvic floor group for managing constipation and fecal incontinence in the elderly. Furthermore, regardless of the state of muscle material properties in the elderly, a comprehensive training approach that includes pelvic floor muscles, rectus abdominis, and erector spinae muscles proves more effective than focusing solely on the pelvic floor muscles in constipation and fecal incontinence rehabilitation trainings.

#### 3.3.3 Comparative analysis of simulated rehabilitation muscles for urinary incontinence and urinary retention

**FIGURE 16:**
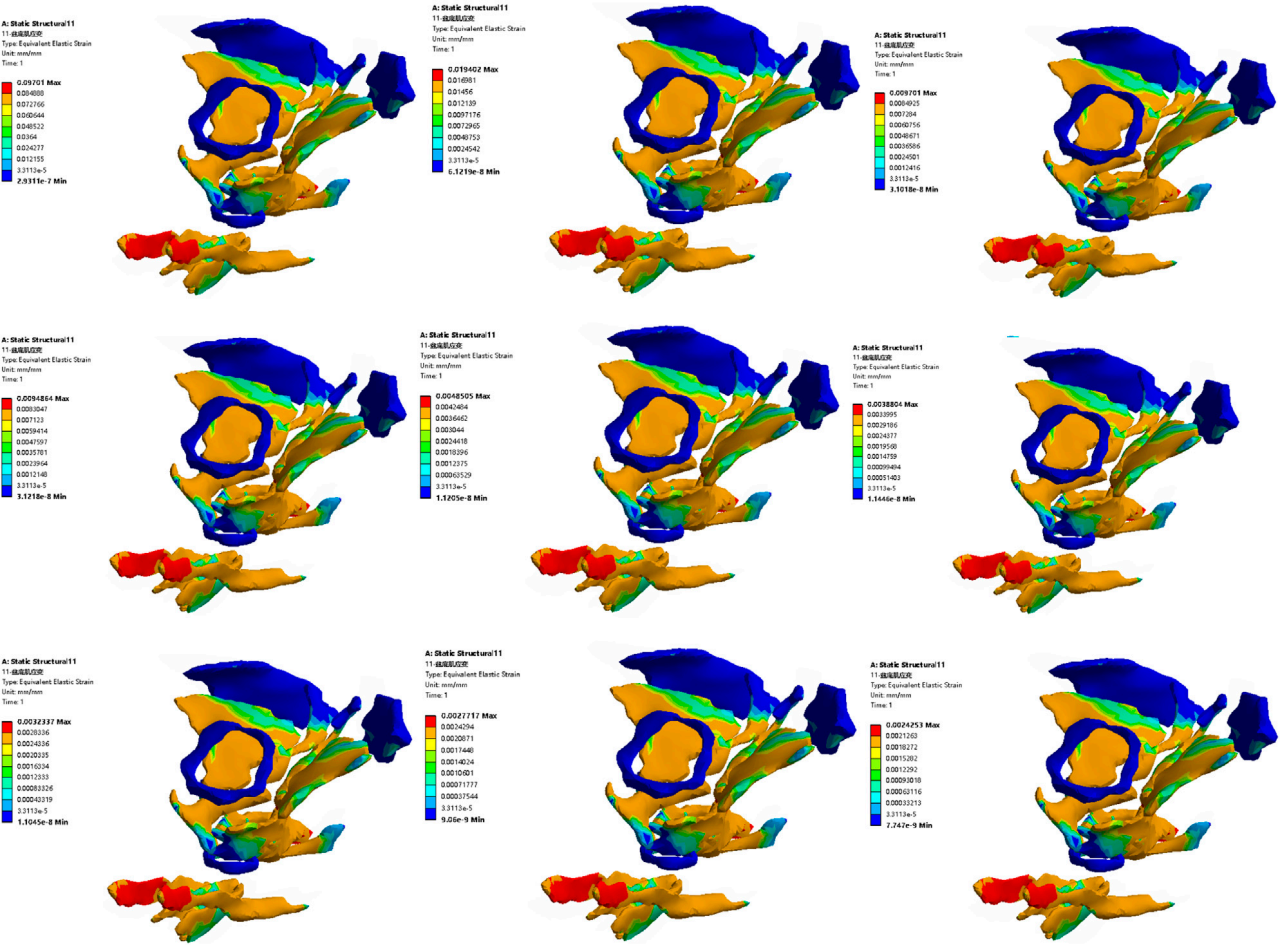
Stress diagram of external anal sphincter in simulation No.6.

**FIGURE 17:**
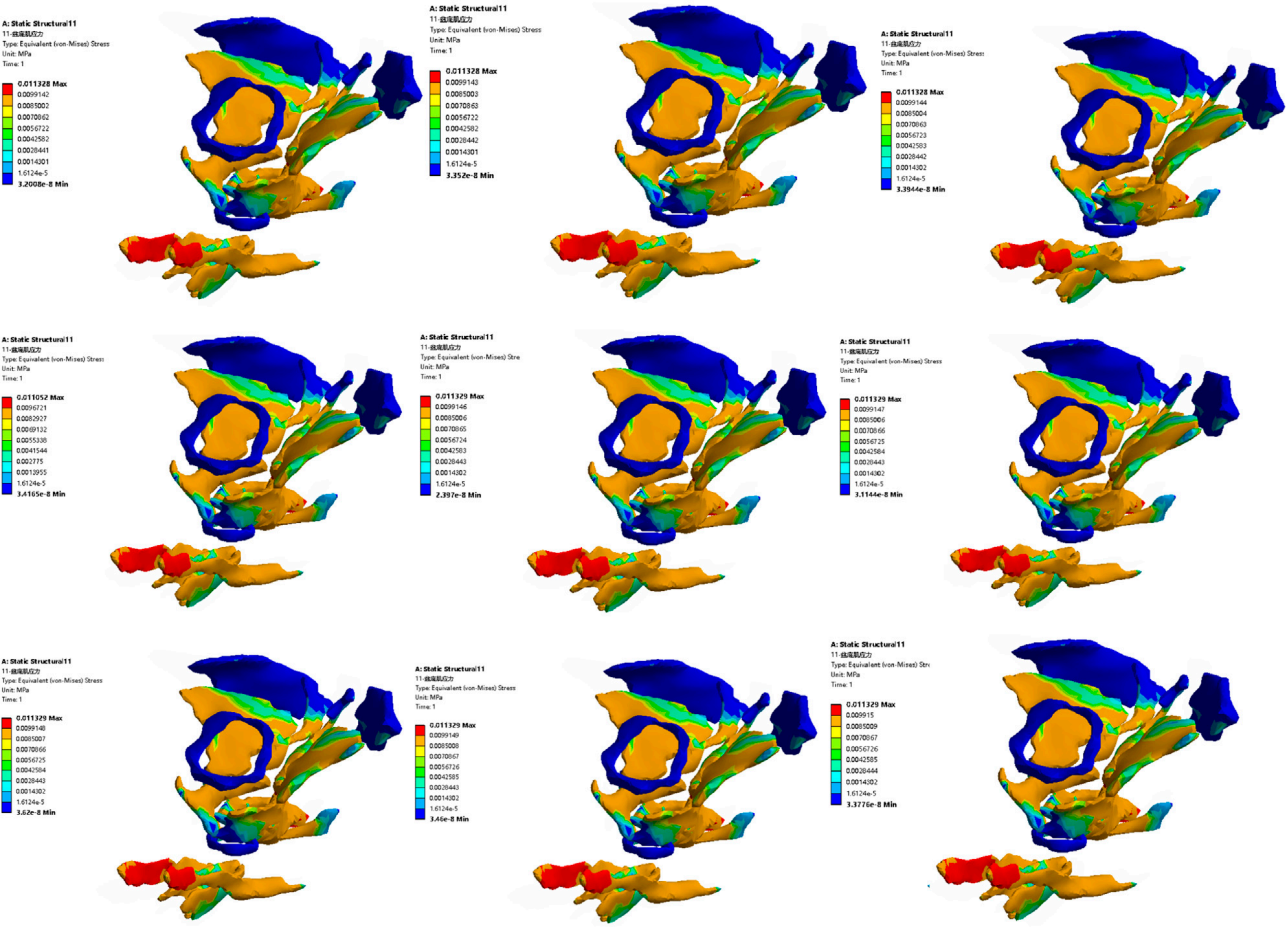
Stress diagram of pelvic floor muscles in simulation No.6.

Simulation plans 3, 4, 6, 8, and 9 were designed to simulate the muscles enhancement and impairment effects of urinary incontinence rehabilitation training. The targeted muscle groups included levator ani muscle, levator ani muscle and urethral sphincter, pelvic floor muscles, pelvic floor muscles and hip muscles, pelvic floor muscles and rectus abdominis muscle and hip muscles and erector spinae muscle. By comparing the degree of improvement in RVA under different simulations, it can be indicated that rehabilitation training progressively changes the material properties of muscles in elderly people from less than normal to normal, the efficacy of training the levator ani muscle alone surpassed that of combined training with the urethral sphincter, and also outperformed the broader group training involving pelvic floor muscles and rectus abdominis muscle and hip muscles and erector spinae muscle, pelvic floor muscles, pelvic floor muscles and hip muscles, sequentially (Figures 10, 11). Furthermore, when the material properties of the muscles from greater than the normal value to the normal value, the training effectiveness of the pelvic floor muscles excelled over that of the pelvic floor muscles and rectus abdominis muscle and hip muscles and erector spinae muscls, levator ani muscles, levator ani muscles and urethral sphincter, and pelvic floor muscles and hip muscles in sequence (Figures 16, 17).

Simulation plans 2, 3, 4, and 6 were designed to simulate the muscles strengthening and impairment effects of urinary retention rehabilitation training. The muscles include urethral sphincter, levator ani muscle, levator ani muscle and urethral sphincter, and pelvic floor muscles. By comparing the degree of improvement in RVA under different simulations, it can be concluded that rehabilitation training gradually changes the material of muscles in elderly people from less than normal to normal, and the training’s efficacy on levator ani muscle surpassed urethral sphincter alone and levator ani muscle combined with the urethral sphincter (Figures 10, 11). The training’s efficacy on levator ani muscle and also exceeded training effects on pelvic floor muscles. In addition, when the material properties of the muscles gradually changes from greater than the normal value to the normal value, the effect of pelvic floor muscles is superior to those involving levator ani muscle alone, or levator ani muscle in combination with urethral sphincter, and urethral sphincter alone respectively (Figures 16, 17).

It should be noted that irrespective of the muscle material properties in the elderly, the training of the levator ani muscle alone demonstrates superior effectiveness over combined training with the urethral sphincter in managing urinary incontinence and retention. Furthermore, both individual and combined training of pelvic floor muscles with rectus abdominis muscles, hip muscles, erector spinae muscles provide more significant benefits compared to training that combines only the pelvic floor muscles with hip muscles.

Moreover, it was observed that alterations in the material properties of muscles from 0.05 to 0.25 times did not significantly impact the ARA. Similarly, when the muscle material properties ranged from 1.50 to 2.00 times, there was no significant change in the RVA.

## 4 Discussion

This study gathered muscles medical imaging from a elderly male to develop and verified a finite element model. This study explored the quantitative relationship between rehabilitation training methods on muscles and urinary and defecation dysfunction, ultimately elucidating the mechanism of improving urinary and defecation dysfunction. One of the advantages of finite element models is that they provide a cost-effective solution for studying complex biomechanical processes, such as rehabilitation training for bowel and bladder functions, without the need for extensive resources, specialists, or equipment. Additionally, these models enhance safety by eliminating the potential risks associated with human trials. Due to the consistent reproducibility of computations, finite element models are able to deliver highly repeatable results. Previous studies have developed some pelvic floor finite element models for the treatment of urinary incontinence, however that predominantly focused on young postpartum women (Hu and Pierre, 2019), leading to a gap in models suited for older men. Male and female pelvic floor anatomy are different, and aging individuals experience physiological changes including decreased muscle strength and changes in pelvic floor tissue relaxation and elasticity. Currently, there is a lack of finite element model of the pelvic floor for analyzing fecal incontinence, constipation, urinary incontinence and urinary retention in elderly men, therefore it is very necessary to construct a finite element model of the pelvic floor of elderly men to guide rehabilitation and treatment. In addition, our model also integrates additional muscular systems, such as those of the back, hip, and abdomen, which play significant roles in urination and defecation rehabilitation training and are often overlooked in existing models. This innovative approach is more conducive to simulating the rehabilitation training methods and measuring the quantitative relationship between five rehabilitation training methods and the urinary and defecation control ability of elderly men, which is also one of the innovative points of this study.

The primary structures that affect the ability to control bowel movements are mainly divided into three parts including pelvic floor muscles, core muscles and sacral nerves. Pelvic floor muscles are crucial for regulating urination, defecation, and maintaining vaginal contractions. Core muscles, encompassing the waist, abdomen, hips, and legs, serve as pivotal points for trunk movements, enhancing stability for the spine and pelvis, and increasing abdominal pressure. Sacral nerves play a vital role in managing the reflexes of the bladder, sphincters, and pelvic floor. In this study, we systematically investigatesd the impact of rehabilitation training on the urinary and defecation abilities of elderly by analyzing the angular changes of ARA and RVA in response to alterations in the material properties of involved muscles.

Regarding the changes in material properties of pelvic floor muscles in the elderly, it is observed that these tissues physiologically lose some elasticity due to aging, potentially reducing the Young’s module of soft tissues (Bhattarai and Staat, 2018). Regular muscle training or other healthy habits in some elderly individuals may increase the Young’s module, potentially reversing some age-related degenerative changes. It is important to note that changes in the Young’s module of pelvic floor muscles in the elderly are not absolute. These changes depend on individual characteristics, natural aging, disease types, and cannot be universally generalized. Hence, our study simulated a range of material properties from 0.05 to 2 times to accommodate various simulation scenarios.

The normal range of ARA and RVA vary, and the variability in results could be attributed to the individual differences among assessors or the participants. According to literature (Xue, 1996; Mortele and Fairhurst, 2007; Colaiacomo et al., 2009; Bitti et al., 2014; Kobi et al., 2018; Zhou and Lai, 2021; The MR Group of the Radiology Branch of the Chinese Medical Association, 2022), the typical resting range of ARA is between 90° and 127°, while during defecation, it extends from 120° to 152.4°. The resting RVA spans from 90° to 120° (Liu et al., 2013). In cases of anterior pelvic dysfunction, RVA values can expand from the normal range of 90°–120°–160°–180°during the tension phases (Fielding et al., 2000). Some research also suggests that RVA for individuals with stress urinary incontinence may exceed 140° (Li and Yan, 2015). The results indicate that as the material properties of muscles or muscle groups change, ARA and RVA are gradually approaching the normal range. Therefore, theoretically, it can be posited that rehabilitation training methodologies including biofeedback, exercise training, electrical stimulation, magnetic stimulation, and vibration training may enhance the ability of elderly men to control bowel movements.

The responsiveness of different muscles to changes in ARA and RVA angles can be evaluated by comparing the slopes of each line in Tables 8 and 9. A higher slope indicates a greater responsiveness of that muscle or muscle combination to changes in ARA and RVA angles. Research findings indicate that individual training of specific muscle groups, particularly the levator ani muscle, proves more effective in treating disorders of both urination and defecation in the elderly compared to combined training with pelvic floor and urethral sphincter muscles. This could be due to the specific role that the levator ani muscle plays in supporting the pelvic organs and controlling the functions closely related to the bowel movements. While the specific research studies that the comparative effectiveness of isolated levator ani muscle training *versus* comprehensive pelvic floor muscle training might not be directly available, general principles of physical therapy and muscle rehabilitation suggest that targeted exercises can indeed improve the functionality of specific muscles more effectively than generalized training. A study reached a similar conclusion to ours, the study pointed out that with the increase of external load, the training effect on certain muscles significantly increases, while the training effect on other muscles is much smaller. The simulation results show that compared with pure weightlifting training, training based on optimal load orientation concept (OLOC) can significantly improve the training effect of specific muscles, and training methods targeting specific muscles maximize the training effect of target muscles (Song et al., 2018).

In addition, research findings indicate that targeted muscle rehabilitation training for the levator ani muscle and anal external anal sphincter in cases of fecal dysfunction, and for the levator ani muscle and pelvic floor muscles in cases of urinary dysfunction, theoretically holds the greatest potential for significant improvement in the urinary and defecation control ability of the elderly. Previous studies (Zhou and Lai, 2021) have highlighted that the key factors influencing individual urinary and defecation control ability include the levator ani muscle, puborectalis muscle, and external anal sphincter, all of which are closely associated with fecal dysfunction. Abnormalities in the structure or function of these muscles lead to bowel control problems. This aligns with the conclusions of our research, which suggests that optimizing the function of the peri-anal muscle group is essential for improving urinary and defecation control ability. Furthermore, for urinary function, the pelvic floor muscles are the most critical source of support (Li and Lu, 2023), with the levator ani muscle playing a significant supportive role (Ashton-Miller and DeLancey, 2007). The literature (Zhou et al., 2020) pointed out that pelvic floor muscle exercise, mainly involving the levator ani muscle, is a method for treating pelvic floor dysfunction diseases. Pelvic floor muscles rehabilitation training, including Kegel exercises, pelvic floor muscle training, biofeedback training, electrical stimulation, and acupuncture, is commonly used in elderly males post-laparoscopic radical prostatectomy (Feng, 2022) and for managing lower urinary tract symptoms post-stroke (Tibaek et al., 2017).

In conclusion, these results are significant for understanding and treating muscle-related urination and defecation dysfunction. The findings emphasize the importance of personalized and targeted rehabilitation approaches in effectively addressing pelvic floor dysfunctions. Some studies (Okada et al., 2001; Tang et al., 2020) have employed shear wave elastography (SWE) to assess the biomechanical properties of pelvic floor muscles. Future research should further explore how specific rehabilitation training methods practically affect muscle material properties, aiming to adjust these properties through tailored exercises to improve bowel disorders in the elderly. When designing and implementing targeted rehabilitation training, it is recommended to advocate for personalized and dynamic treatment plans. Specifically, the response characteristics of different muscles under varying stress conditions should be considered, and training intensity and muscle group rehabilitation strategies should be adjusted based on the changes in muscle material properties to enhance the effectiveness of rehabilitation training for the elderly.

The study acknowledges several limitations. Firstly, due to constraints, this study was conducted on a single elderly male subject, and the results may not fully represent all elderly men. Secondly, the complexity of the human body necessitated the simplification of certain biomechanical processes. This includes a focus on specific muscle groups, such as the abdominal muscles specifically refer to the rectus abdominis, and the hip muscles specifically refer to the iliopsoas, quadriceps, gluteus maximus, hamstrings, gluteus medius, and adductor longus The back muscles were specifically the erector spinae, without incorporating ligament roles or all muscle combinations. Third, while the study provides insights into muscle functionality and its effects on rehabilitation outcomes, the results of this study are based on the relationship between muscles and ARA, RVA, and it does not a direct relationship between rehabilitation training methods and urinary and defecation control ability. This possibly leading to over-idealized conclusions. In actual rehabilitation training, it may not be possible to train a single muscle alone, and it is also difficult to achieve proportional changes in muscles. However, this research offers a methodological approach to explain the mechanism of urinary and defecation control ability and elucidates the potential mechanisms linking rehabilitation training methods with the urinary and defecation control ability in the elderly.

## 5 Conclusion

In conclusion, by constructing and verificating a finite element model the study demonstrates that rehabilitation training, including exercise training, electrical stimulation, magnetic stimulation, biofeedback, and vibration stimulation, can improve the urinary and defecation control ability of elderly men. Crucially, the research results highlights the pivotal role of the levator ani muscle, external anal sphincter, and pelvic floor muscles in the managing of urination and defecation dysfunction. Moreover, this study emphasizes the effectiveness of targeted rehabilitation training in restoring urinary and defecation control among the elderly, emphasizing the need to adjust muscle training intensity and method based on the individual’s muscle material properties to optimize therapeutic outcomes.

## Data Availability

The raw data supporting the conclusion of this article will be made available by the authors, without undue reservation.
